# Novel Phenanthrene-Degrading Bacteria Identified by DNA-Stable Isotope Probing

**DOI:** 10.1371/journal.pone.0130846

**Published:** 2015-06-22

**Authors:** Longfei Jiang, Mengke Song, Chunling Luo, Dayi Zhang, Gan Zhang

**Affiliations:** 1 State Key Laboratory of Organic Geochemistry, Guangzhou Institute of Geochemistry, Chinese Academy of Sciences, Guangzhou, 510640, China; 2 Lancaster Environment Centre, Lancaster University, Lancaster, LA1 4YQ, United Kingdom; 3 Graduate University of Chinese Academy of Sciences, Beijing, 100039, China; University of Kansas, UNITED STATES

## Abstract

Microorganisms responsible for the degradation of phenanthrene in a clean forest soil sample were identified by DNA-based stable isotope probing (SIP). The soil was artificially amended with either ^12^C- or ^13^C-labeled phenanthrene, and soil DNA was extracted on days 3, 6 and 9. Terminal restriction fragment length polymorphism (TRFLP) results revealed that the fragments of 219- and 241-bp in *Hae*III digests were distributed throughout the gradient profile at three different sampling time points, and both fragments were more dominant in the heavy fractions of the samples exposed to the ^13^C-labeled contaminant. 16S rRNA sequencing of the ^13^C-enriched fraction suggested that *Acidobacterium* spp. within the class *Acidobacteria*, and *Collimonas* spp. within the class *Betaproteobacteria*, were directly involved in the uptake and degradation of phenanthrene at different times. To our knowledge, this is the first report that the genus *Collimonas* has the ability to degrade PAHs. Two PAH-RHD_α_ genes were identified in ^13^C-labeled DNA. However, isolation of pure cultures indicated that strains of *Staphylococcus* sp. PHE-3, *Pseudomonas* sp. PHE-1, and *Pseudomonas* sp. PHE-2 in the soil had high phenanthrene-degrading ability. This emphasizes the role of a culture-independent method in the functional understanding of microbial communities *in situ*.

## Introduction

Polycyclic aromatic hydrocarbons (PAHs) can enter the environment from both anthropogenic and natural sources. They are primarily derived from the incomplete combustion of organic matter at high temperatures. In addition, PAHs can be released into the environment through natural processes such as forest fires, and direct biosynthesis by microbes and plants [1]. Their presence in the environment poses a considerable threat to public health and ecosystems because of their acute toxicity, potential mutagenicity, and carcinogenicity. This has led to their classification as priority pollutants by the U.S. Environmental Protection Agency [2].

The contamination resulting from PAHs can be removed by microorganisms, as has been shown for other pollutants. The role of bacteria in the degradation of organic pollutants has attracted much attention. A successful bioremediation strategy involves either encouraging the native microbial community to rapidly degrade the pollutants, or the addition of more-effective degradative microbes, in the absence of a native degrading biota [3]. The identification of pollutant-degrading populations within microbial communities is therefore essential for the design, control, and optimization of the bioremediation of PAHs-contaminated aquifers. Traditional isolation/cultivation studies have provided most of our knowledge regarding how pollutants are biodegraded under artificial conditions and the PAHs degradation pathways. The genes associated with the degradation process have been identified, with particular emphasis given to the PAHs ring hydroxylating dioxygenase (PAHs-RHD) [4, 5]. However, it has proven difficult to obtain a pure culture of target bacteria in the laboratory, with only a small percentage of the organisms found in soil communities capable to be cultured, which does not reveal the real picture within a specific site. Furthermore, inoculation cannot always achieve an ideal remediation, because of the lack of understanding of where the target microorganisms are located within the overall biota [6]. In recent years, culture-independent methods, such as stable-isotope probing (SIP), have been used to link the growth of microbial populations to specific metabolic processes. This has been achieved by feeding microorganisms a substrate containing an isotope to label the microbial DNA, allowing the identification and characterization of novel organisms that previously escaped detection. The ^13^C-labeled DNA is subsequently separated from ^12^C-DNA on density gradients, and analyzed by molecular techniques such as high-throughput sequencing, terminal restriction fragment length polymorphism (TRFLP) and denaturing gradient gel electrophoresis (DGGE), for phylogenetic characterization. Microbial populations within complex communities that are responsible for the degradation of targeted contaminants can then be identified [7–9]. Besides DNA, RNA and phospholipid fatty acid (PLFA) can also be used as biomarkers in a fingerprint analysis [10, 11]. To date, SIP has been successfully used to identify a variety of bacteria that assimilate diverse compounds [9, 12–18].

Phenanthrene was used as a model PAHs in the present study due to its ubiquity in nature and the fused-ring structure resembling higher-molecular-weight PAHs that are known to be carcinogenic. To date, SIP studies undertaken on PAHs biodegradation have been limited to previously contaminated media such as coal-tar polluted sediments [19], road runoff polluted soils [20, 21], and polluted soils from a former gas-manufacturing plant [22–24], former wood preserving facility [25], former coking plant site [5], and a tarmacadam-producing plant [18]. However, SIP has not yet been used to investigate potential PAHs degraders in non-contaminated soils. It is well-known that bacterial diversity can usually be repressed by the existence of toxic organic pollutants [26–28]. Hence, unpolluted forest soils are likely to have a more varied bacterial community than contaminated soils, which could be quite different from the community in contaminated sites. Identification of the functional community responsible for the degradation of phenanthrene is important for understanding the biogeochemistry of PAHs in natural soils, and the clean-up ability of the soil itself. In this study, ^13^C-DNA targeted SIP was applied to a clean forest soil to investigate phenanthrene degradation therein, and to determine the organisms responsible for this *in situ* degradation. Traditional isolation and pure culture techniques were also used for comparison with the effectiveness of SIP. The aim was to achieve a full understanding of phenanthrene degradation in this ‘clean’ medium.

## Materials and Methods

### Development of phenanthrene-degrading microcosms

Soil samples were collected from a forest in Luoji Mountain (27°34′N, 102°25′E, altitude 2200 m), Sichuan Province, China. The research site is owned by Guangzhou Institute of Geochemistry. The field studies did not involve endangered or protected species and no specific permits were required for the described field studies.The soil (pH = 5.5) had a high organic matter content (6.2%), and was classified as a sandy loam soil based on the texture analysis. In laboratory, the samples were homogenized, sieved through a 4 mm screen and stored at 4°C until use.

A sample of 6 g soil (dry weight) was placed in a 150 mL serum bottle containing 20 mL phosphate-buffered mineral medium [29]. The bottles were sealed with rubber stoppers and compressed with an aluminum seal. After sealing, ^12^C-labeled phenanthrene (99%, Cambridge Isotope Laboratories, Inc., Tewksbury, MA, USA) or ^13^C-labeled phenanthrene (^13^C_6_-PHE, 99%, Cambridge Isotope Laboratories, Inc.) was added to the bottles via a gas-tight syringe, to a final phenanthrene concentration of 1 mg/kg. The serum bottles were opened each day, kept in the air for 1 hour to ensure that the oxygen in the microcosms remained close to environment and resealed. The treatments included: no phenanthrene controls, autoclaved controls, and ^12^C- and ^13^C-labeled phenanthrene samples. Six samples were prepared for each treatment. The cultures were incubated at 20°C, with reciprocal shaking. On days 3, 6, and 9 after incubation, two samples from each treatment were sacrificed for phenanthrene analysis and DNA extraction.

### Phenanthrene measurement

On days 3, 6, and 9 after incubation, two samples from each treatment were used for phenanthrene analysis as follows. A freeze-dried soil sample was homogenized, pulverized, spiked with 1,000 ng of deuterated PAHs as a surrogate standard and extracted with dichloromethane (DCM) in a Soxhlet apparatus for 48 h with activated copper added to remove the sulfur. The extract was concentrated to about 0.5 mL after a solvent exchange with hexane. The soil extracts were purified with a silica gel/alumina column (8 mm i.d.) filled with anhydrous Na_2_SO_4_ (1 cm), neutral silica gel (3 cm, 3% deactivated) and neutral alumina (3 cm, 3% deactivated) from top to bottom, with an eluent of 15 mL hexane/DCM (1:1, v/v). After being concentrated to approximately 50 μL using a gentle stream of N_2_, 1,000 ng of hexamethylbenzene as an internal standard was added to all samples prior to instrumental analysis.

Phenanthrene was analyzed by gas chromatography (model 7890, Agilent, Santa Clara, CA, USA), using a capillary column (DB-5MS, 30 m, 0.25 mm, 0.25 μm) and a mass spectrometric detector (MSD, model 5975, Agilent). Samples (1 μL) were injected under splitless mode with a 10 min solvent delay time. High-purity helium was used as a carrier gas with a flow velocity of 1.83 mL/min. The temperature of the injector and transfer line were 290°C and 300°C, respectively. The initial oven temperature was set at 60°C for 1 min and raised to 290°C at a rate of 3°C/min, and then held for 20 min.

### DNA extraction and ultracentrifugation

DNA extraction was conducted on two soil samples from each treatment that were incubated for 3, 6, and 9 days, using a Powersoil DNA extraction kit (MO BIO Laboratories, Inc. Carlsbad, CA, USA) following the manufacturer’s instructions. The DNA content was quantified with an ND-2,000 UV-Vis spectrophotometer (NanoDrop Technologies, Wilmington, DE, USA). Thereafter, about 10,000-ng DNA was added to Quick-Seal polyallomer tubes (13 × 51 mm, 5.1 mL, Beckman Coulter, Pasadena, CA, USA) and spun in Tris-EDTA (TE, pH 8.0)/CsCl solution. Prior to sealing the tubes with one cordless quick-seal tube topper (Beckman Coulter), the average buoyant density (BD) of all prepared gradients was confirmed with a digital refractometer (model AR200, Leica Microsystems Inc., Buffalo Grove, IL, USA), and adjusted by adding small volumes of CsCl solution or Tris-EDTA buffer. The tubes were transferred to an ultracentrifuge (Optima L-100XP, Beckman Coulter). Centrifugation was performed at 45,000 g (20°C) for 48 h. Subsequently, the centrifuge tubes were placed onto a fraction recovery system (Beckman Coulter) and fractions (150 μL for each) were collected. The BD of each fraction was then measured, and CsCl was removed by introducing concentrated ethanol [30].

### PCR and TRFLP

The ultracentrifugation fractions were subjected to a polymerase chain reaction (PCR). The 16S rRNA PCR primers (Operon Biotechnologies, Bangalore, India) were 27F-FAM (5’-AGAGTTTGATCMTGGCTCAG, 5’ end-labeled with carboxyfluorescine) and 1492R (5’-GGTTACCTTGTTACGACTT), and the PCR amplifications were conducted in a Peltier thermal cycler (BIO-RAD, Hercules, CA, USA) using a protocol adopted previously: 94°C (5 min); 94°C (30 s), 55°C (30 s), 72°C (1.5 min) (30 cycles); and 72°C (5 min) [8]. The presence of PCR products was confirmed by 1% agarose gel electrophoresis and the subsequent staining of the gels with ethidium bromide. PCR products were further purified with an Omega ENZA Cycle-Pure kit (Omega Bio-Tek, Inc., Norcross, GA, USA). The purified PCR products from the total DNA and fractions were digested with *Hae*III, following the manufacturer’s instructions (New England Biolabs, Ipswich, MA, USA). One nanogram of labeled PCR product was run on a genetic analyzer (ABI 3730, Applied Biosystems, Foster City, CA, USA) with Peak Scanner Software v. 1.0 (Applied Biosystems), and using ROX500 as the internal standard to separate the DNA fragments. Data was analyzed with Peak Scanner Software v. 1.0. The percentage abundance of each fragment was determined as described previously [30].

### Detection of the PAH-RHD_α_ gene in microcosms

The presence of the PAH-RHD_α_ gene was investigated using both the PAH-RHD_α_ GP and GN primer pairs on the heavy DNA fractions. A gradient PCR was performed with annealing temperatures ranging from 52 to 62°C [31]. However, only the PAH-RHD_α_ GP primers 641F (5’-CGGCGCCGACAAYTTYGTNGG) and 933R (5’-GGGGAACACGGTGCCRTGDATRAA) produced a strong and specific amplicon and were therefore selected for the study. Further amplification reactions were conducted in a volume of 50 μL, as reported in a previous study: 95°C (5 min); 95°C (30 s), 54°C (30 s), 72°C (30 s) (30 cycles); and 72°C (7 min) [31].

### Sequencing of 16S rRNA genes and the partial PAH-RHD_α_ gene

Clone libraries of the 16S rRNA genes were constructed with the ^13^C fraction of ^13^C-labeled DNA, amplified using a previously adopted protocol, where 27F-FAM was replaced by 27F [8]. In addition, amplicons generated with PAH-RHD_α_ primer pairs were also prepared for cloning and sequencing. Purified PCR products were cloned into *Escherichia coli* JM109 using a TA cloning kit (TaKaRa Biotechnology, Inc., Shiga, Japan). *E*. *coli* clones were grown on Luria-Bertani (LB) medium, solidified with 15 g agar/L in the presence of 50 μg/L ampicillin for 16 h at 37°C. The positive single colonies were isolated and cultivated in the LB medium containing 50 μg/L ampicillin overnight. Colonies with inserts were verified by PCR with primers M13 forward (5’-TGTAAAACGACGGCCAGT-3’) and M13 reverse (5’-AACAGCTATGACCATG-3’), and the plasmids were extracted with an EZNA Plasmid Mini kit (Omega Bio-Tek, Inc.). The insertions were sequenced with a genetic analyzer (ABI 3730, Applied Biosystems), using the pair of M13 universal primers. The sequences obtained were compared with the GenBank database, using the Basic Local Alignment Search Tool (BLAST) algorithm (National Center for Biotechnology Information, Bethesda, MD, USA). Phylogenic trees for the partial PAH-RHD_α_ sequences along with the closest matches in GenBank were obtained by the neighbor-joining method using Molecular Evolutionary Genetics Analysis, version 5.0 (MEGA 5.0).

The 16S rRNA gene clone libraries and the partial PAH-RHD_α_ gene sequences determined in this study were deposited under accession numbers KF035825 and KF035826 for partial 16S rRNA gene sequences, and KF656718 and KF656719 for partial PAH-RHD_α_ gene sequences.

### Isolation and identification of phenanthrene-degrading bacteria

The isolation of the phenanthrene degrading microorganisms that were capable of degrading phenanthrene in soils, and their pure cultivation, was attempted. Soil samples (10 g) were suspended in 100 mL mineral salts medium (MSM) (NaCl 0.5 g/L, (NH_4_)_2_SO_4_ 1.0 g/L, K_2_HPO_4_ 1.5 g/L, KH_2_PO_4_ 0.5 g/L, MgSO_4_·7H_2_O 0.2 g/L, pH = 7.0), with 1- and 10-mg/kg (MSM-C1 and MSM-C10) phenanthrene as the sole carbon source, which corresponded to 1- and 10-times the phenanthrene used in the SIP study, respectively. The inoculum was incubated at 30°C at 180 rpm for 4 days. Subsequently, 100 μL of the culture was subcultured in 100 mL fresh MSM-C1 and MSM-C10 respectively, and then incubated under the same conditions for another 4 days. After 3 sequential rounds of enrichment, the cultured medium was diluted 10–10^5^-fold with sterilized water, and then daubed on a solid mineral salts medium supplemented with 1 and 10 mg/L phenanthrene, respectively. Single colonies were selected and cultured in MSM-C1 or MSM-C10 for 4 days, and the degrading capacities of the isolates were tested in liquid culture by adding 3- or 30-μg phenanthrene to 3-mL cell suspensions. Cultures were incubated in the dark at 180 rpm at 30°C. The degradation efficiency was determined after 3 days using the method described above. Genomic DNA was extracted and 16S rRNA was amplified by PCR using the primers 27F and 1492R as above. Cultivable phenanthrene degraders were identified by 16S rRNA sequencing, and the sequences of the isolates were deposited in the GenBank database under accession numbers KF035827 to KF035829.

## Results and Discussion

Previous SIP studies regarding the biodegradation of PAHs focused primarily on samples from formerly contaminated sites. To expand on this, this study applied SIP to a clean forest soil, and therefore fresh insights on the biogeochemistry of PAHs in natural soils were anticipated.

### Biodegradation of phenanthrene in soil

As shown in Table 1, the phenanthrene concentration remained constant from day 3 to 9 in the sterile soils. In contrast, significant phenanthrene biodegradation was observed after 3 days of incubation in the unsterilized microcosms. Phenanthrene in the unsterilized soils degraded quickly, with 70% degradation achieved after 9 days. Throughout the process, no significant difference (*p* >0.05) was found between soils amended with ^13^C- and ^12^C-labeled phenanthrene.

**Table 1:** Ranges for percentage of phenanthrene remaining in soil-liquid slurries over time.

Time (days)	Sterile controls	^12^C- phenanthrene amended samples	^13^C- phenanthrene amended samples
**3**	85–90	66–72	64–75
**6**	83–87	40–45	42–48
**9**	86–89	30–36	26–35

Phenanthrene is a widespread pollutant that biodegrades more rapidly than many other organic pollutants [32]. The degradation rate observed in this study was comparable with results reported previously [5, 21, 22], but considerably higher than the rates reported by Renzt et al. and Powell et al [33, 34]. This may be attributed to the soil properties, experimental conditions such as the laboratory microcosm versus field scale conditions, and the concentration and composition of contaminants in the system tested. It was found that when phenanthrene co-existed with other PAHs, such as naphthalene and pyrene, it degraded at a slower rate than when present alone, which was explained by potential interactive effects, the toxicity, and both non-competitive and competitive inhibition [34]. In addition, soils used in laboratory experiments are usually homogeneous and the growth conditions tend to be optimized, which enhances the degradation capacity compared with the heterogeneous natural soil in the field.

### TRFLP results for SIP

**Fig 1:**
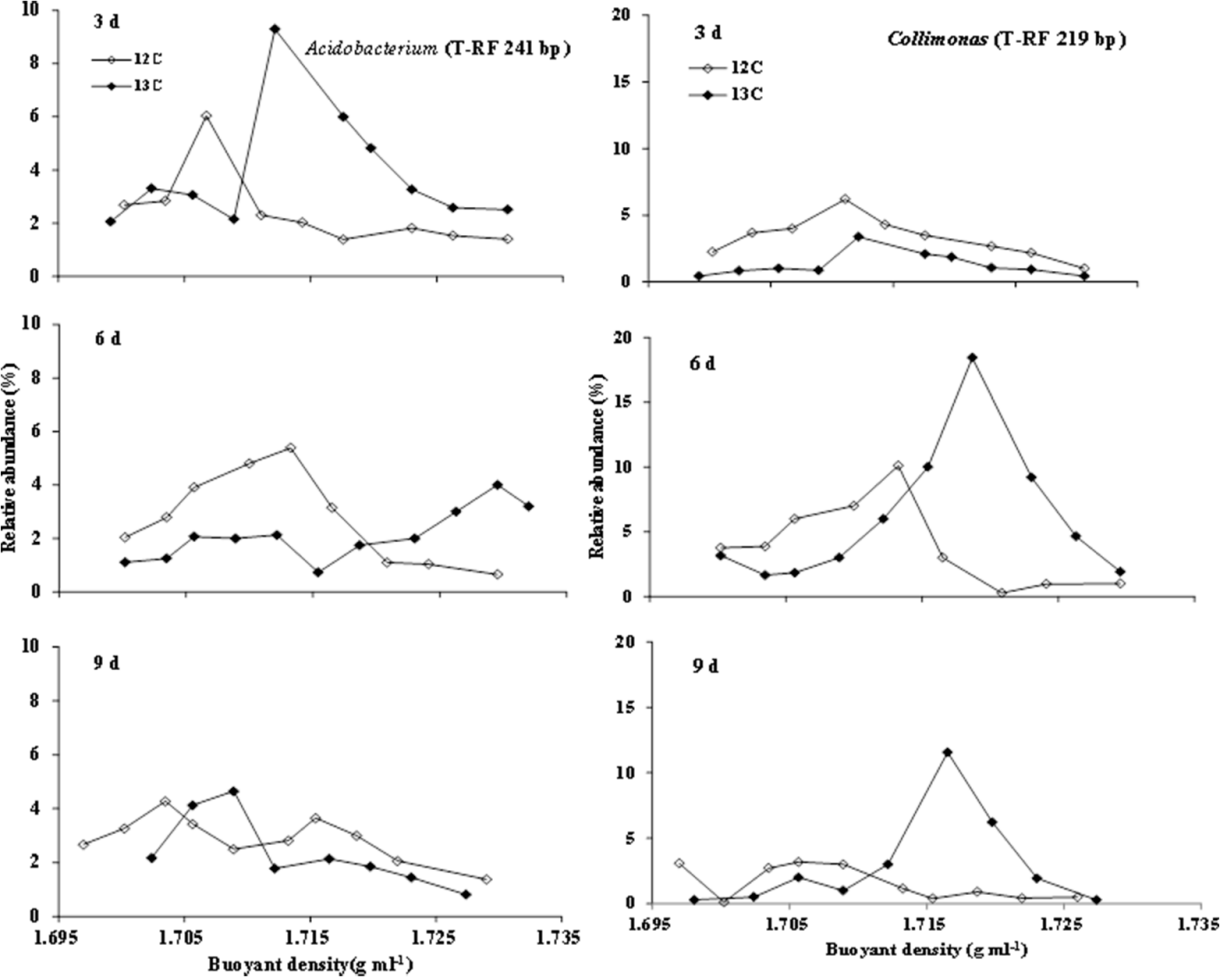
The shift trendency of 241-bp and 219-bp fragments. The relative abundance of the dominant 241-bp and 219-bp fragments over a range ofbuoyant density (BD) from DNA extracted after 3, 6, 9 days from the soil added with either ^13^C or ^12^C labeled phenanthrene.

The organisms responsible for ^13^C assimilation were identified by a comparison of the relative abundances of specific terminal restriction fragments between the control (amended with ^12^C-labeled phenanthrene) and the sample (amended with ^13^C-labeled phenanthrene), at selected time points for each fraction. Based on the TRFLP results, the fragment 241-bp *Hae*III TRF displayed a clear trend of increased relative abundance at higher buoyant densities in samples amended with ^13^C-phenanthrene compared to the ^12^C-phenanthrene treatment on days 3 and 6. This indicated that ^13^C was incorporated by the microorganisms represented by these fragments (Fig 1). However, this trend was not evident on day 9. For fragment 219-bp *Hae*III TRF, an increased relative abundance at higher buoyant densities in samples amended with ^13^C-phenanthrene compared to the ^12^C-phenanthrene control was observed on days 6 and 9 (Fig 1), but not on day 3. These results may indicate that organisms represented by fragments 241- and 219-bp played a role at different stages of phenanthrene degradation, whereas in the first 3 days the organisms represented by 241-bp played a major role, and by day 9, their role was replaced by organisms represented by the 219-bp band.

### Phylogenetic identification of ^13^C-labeled phenanthrene degraders

Fragment lengths from enzyme digestion were compared to those obtained from in silico digests of 16S rRNA sequence data from ^13^C fractions, to determine the clones they represented. Of the 50 clones sequenced, 21 and 17 clones had restriction enzyme cut sites that matched the TRFLP results for fragments 241- and 219-bp, respectively. The slight difference (2–3 bases) between the measured fragment lengths and those predicted using sequence data has been noted by others [8, 10, 30, 35, 36]. On the basis of the comparative analyses of 16S rRNA genes in this study, the phenanthrene degrading microorganisms represented by 241- and 219-bp were classified as *Acidobacterium* sp. within the class *Acidobacteria*, and *Collimonas* sp. within the class *Betaproteobacteria*, respectively.

The phylum *Acidobacteria* was first identified in 1997 [37], and accounts for around 20% of the soil bacterial community, with large genetic and metabolic diversity in environmental samples [38–41]. *Acidobacterium* species have previously been linked with the degradation of both natural and anthropogenic organic compounds, including petroleum in a coastal stream and beach sediment [42, 43], PAHs in coal-tar contaminated soil [44], landfill soil [45] the rhizosphere [46], and polychlorinated biphenyls (PCBs) from contaminated sediment and soil [45, 47, 48]. Some species in this genus were shown via SIP to be actively involved in the oxidation of propionate [49], and the biodegradation of biocides [50], herbicides [51] and benzene [17]. However, this genus has not been previously linked with phenanthrene degradation; thus uncertainty remains as to whether these microbes are directly involved in the degradation of PAHs, either *in situ* or in a mixed culture. Our results provide strong evidence that microorganisms in this genus are the primary organisms responsible for the degradation of phenanthrene in the mixed community of a forest soil.

The genus *Collimonas* was first described in 2004, with the remarkable characteristic of growing at the expense of living fungal hyphae [52, 53]. An important role of the bacterial genus *Collimonas* in mineral weathering for forest soils has been reported [54, 55]. However, studies reported its ability to degrade organic pollutants [56], that the isolated *Collimonas* sp. is an alkane degrader, and its 16S rRNA sequence is similar to a naphthalene-degrading strain originally classified as *Herbaspirillum* sp.

To our knowledge, this is the first study to use a DNA-based SIP technique to link the phenanthrene-degrading ability of these two genera in a real environment, although a number of studies have been undertaken to identify phenanthrene degrading microbes using SIP [9, 18–25, 34]. This result improves our understanding of the ability of *Acidobacterium* and *Collimonas* species to degrade PAHs pollutants.

### Occurrence of PAH-RHD_α_ functional genes in the microcosm

**Fig 2:**
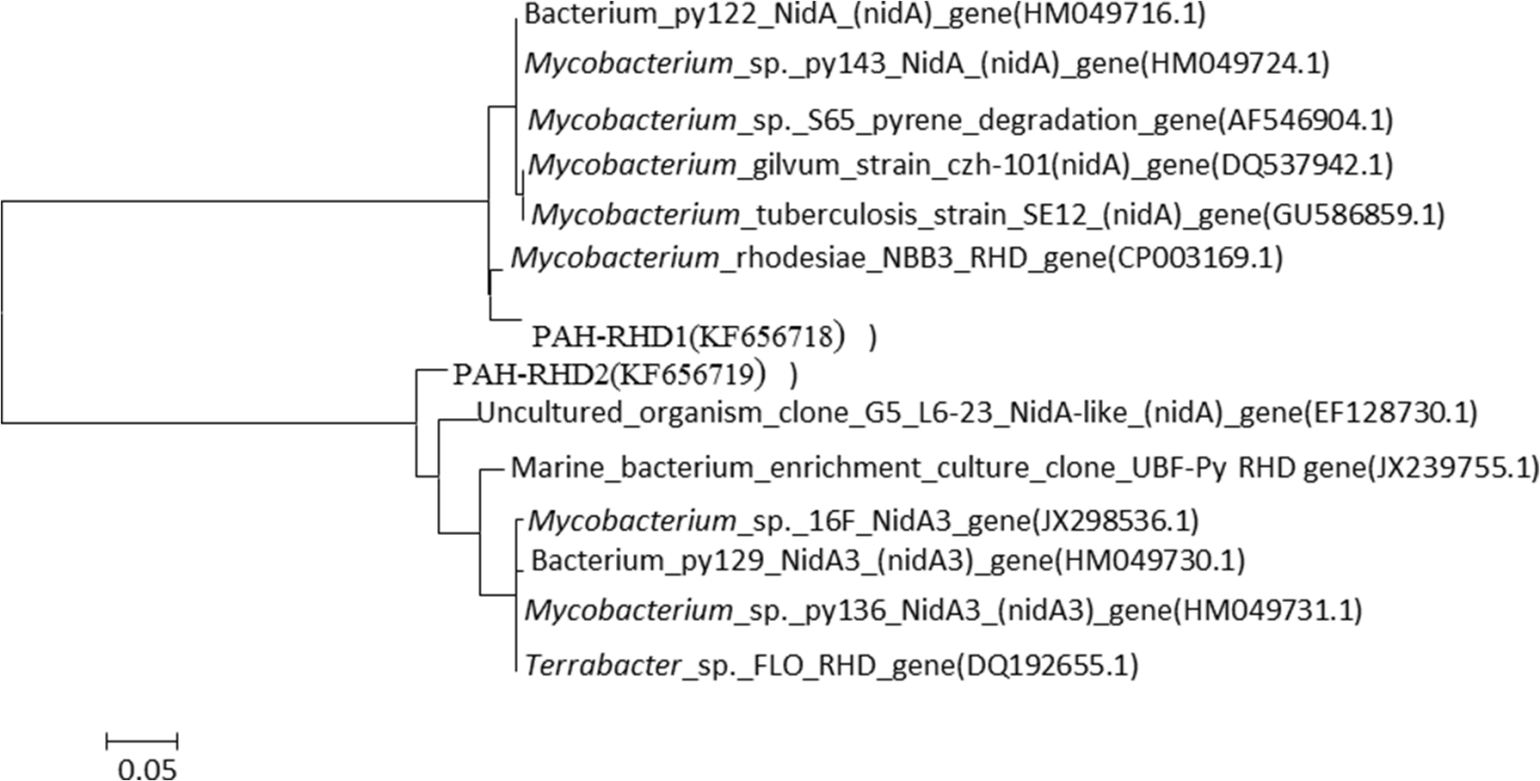
Phylogenetic tree of PAH-RHD_α_ gene. Phylogenetic relationship of PAH-RHD_**α**_ gene cloned from soil treated by 1 mg/kg phenanthrene. PAH-RHD1 and PAH-RHD2 showed 97% and 92% similarity with genes from *Mycobacterium_rhodesiaee* and uncultured organism, respectively.

PAH-RHD_α_ genes from Gram-positive (GP) and Gram-negative (GN) bacteria were analyzed in heavy DNA fractions. On the global scale, the proportion of PAH-RHD_α_ GN tends to be higher than that of the PAH-RHD_α_ GP [31]. However, in the present study only two types of PAH-RHD_α_ GP gene were detected in the heavy DNA, and were affiliated to PAH-RHD_α_ genes from *Mycobacterium rhodesiae* NBB3 (CP003169) and an uncultured organism (EF128730.1) (Fig 2). These may be the functional genes associated with the phenanthrene-degrading strains of *Acidobacterium* and *Collimonas* identified by SIP. It should be noted that the active phenanthrene degrading bacteria might contain other functional genes that could not be targeted by the primer used in the present study.

### Phenanthrene-degrading bacteria derived from isolation and pure culture

**Fig 3:**
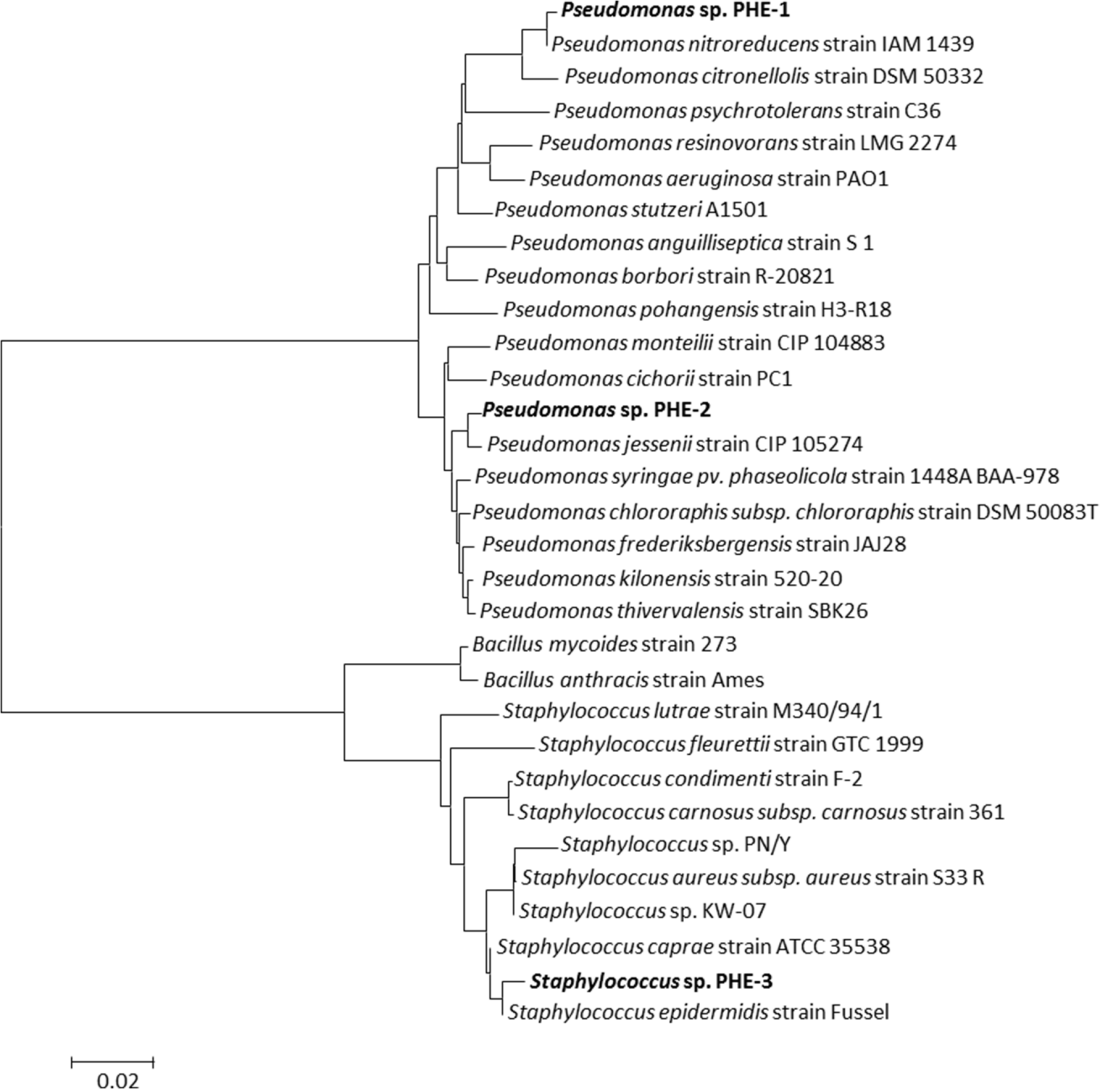
Phylogenetic tree of the isolated phenanthrene degraders. Phylogenetic tree for the taxonomic location of *Pseudomonas* sp. PHE-1, *Pseudomonas* sp. PHE-2 and *Staphylococcus* sp. PHE-3. This tree based on 16S rRNA sequence was produced by MEGA 5.0.

Among the phenanthrene-degrading bacteria using MSM with 1 mg/L and 10 mg/L (w/v) phenanthrene as the sole carbon source, three colonies were selected based on the size of the clear zone in the phenanthrene layer, respectively. DNA sequence analysis identified these three isolates as *Pseudomonas* sp. PHE-1, *Pseudomonas* sp. PHE-2, and *Staphylococcus* sp. PHE-3 (Fig 3). As shown in S1 Table, for the degradation of 1 mg/kg phenanthrene, the most efficient strain was *Pseudomonas* sp. PHE-1 (98.5% degradation within three days), followed by *Pseudomonas* sp. PHE-2 and *Staphylococcus* sp. PHE-3, with 82.3% and 75.6% degradation in the same time period, respectively. At 10 mg/kg phenanthrene, a 51.2% degradation performance was evident in the presence of *Pseudomonas* sp. PHE-1.

*Pseudomonas* sequences are common in PAHs-contaminated soils, and many PAHs degrading isolates are found in this genus. The pathways used by this genus in the degradation of low-molecular-biomass PAHs, including naphthalene and phenanthrene, have been investigated extensively [57]. In addition, the genetic control of the pathways involved in naphthalene degradation by this genus has been studied in detail, and the nucleotide sequences of the genes encoding the upper pathway enzymes in some *Pseudomonas* strains have been clarified [58]. In this study *Pseudomonas* sp. PHE-1 and *Pseudomonas* sp. PHE-2 displayed 99% similarity with *Pseudomonas nitroreducens* and *Pseudomonas jessenii*. However, no enrichment of the appropriate *Pseudomonas* TRF was seen in the ^13^C-labeled fractions, indicating that these microbes were not likely to be the major degraders *in situ*.

Although the genus *Staphylococcus* is not as common as *Pseudomonas* in PAHs-contaminated soils, a few species in this genus have been found to degrade phenanthrene [59, 60]. The species, *Staphylococcus warneri*, has been successfully used to build a consortium with *Bacillus pumilus* capable of efficient degradation of phenanthrene, pyrene, and benzoanthracene [61]. A novel phenanthrene assimilation pathway involving 2-hydroxy-1-naphthoic acid was found in *Staphylococcus* sp. PN/Y, which provided new insight into the microbial degradation of PAHs [59]. By analyzing the 16S rRNA sequence, it was found that *Staphylococcus* sp. PHE-3 displayed 96% similarity to *Staphylococcus* sp. PN/Y and 97% similarity to *Staphylococcus* sp. KW-07. To our knowledge, this is the third species of the *Staphylococcus* genus displaying an ability to degrade phenanthrene.

### Implications for the Understanding of PAHS Biogeochemistry in Natural Soils

These results illustrate the importance of culture-independent approaches compared to culture-dependent approaches for understanding the functions of mixed cultures. Biodegradation has been extensively studied for many decades, and has become a popular option for the elimination of toxic organic pollutants in air, water, and soil media because of its efficacy, and environmentally sustainable and economical characteristics. Phenanthrene can be degraded by some strains under aerobic conditions, and many successful field remediation cases have been reported for PAHs-contaminated sediments and soils. With the aid of advanced methods, if these bacteria can be isolated and cultured under normal laboratory conditions, the pathway adopted by *Acidobacterium* and *Collimonas* in phenanthrene degradation could be clarified. This would provide a new perspective on the biogeochemistry of phenanthrene in natural soils, and a suitable method for the biodegradation of phenanthrene in contaminated environments.

Strain inoculation as a superior method is used in the remediation of sites contaminated with organic pollutants. In this study, the dramatically different results obtained from the isolation and SIP provides some insight into why some artificial constructed systems in the field are not as efficient as expected. This could be a consequence of the intensive competition between native organisms and the inoculated strains. With the aid of the culture-independent SIP technique, the ecological roles of the functional microbes in a specific biota can be identified and characterized. Further artificial manipulation and optimization could stimulate the functional strains; hence, the maximum degradation efficiency would be achieved.

## Supporting Information

S1 Fig Phylogenetic tree of the unclutured degraders. Phylogenetic tree for the taxonomic location of the bacteria corresponding 219- and 241-bp TRFs. The tree is based on 16S rDNA sequence and produced by MEGA 5.0. 2.
(PDF)

S2 Fig The total DNA distribution in different buoyant desity. The density distribution of total DNA in the micocosms amended with unlabled (^12^C) or labled (^13^C) PHE after centrifugation on days 3, 6 and 9.
(PDF)

S1 Table Phenanthrene degradation of isolates at 1 and 10 mg/kg in liquid media (DOCX)

S2 Table Primer pares used in the present study. (DOCX)

S3 Table Numerical data to Fig 1 for T-RF 241bp (DOCX)

S4 Table Numerical data to Fig 1 for T-RF 219bp. (DOCX)

S5 Table Numerical data to Table 1. (DOCX)

S6 Table Numerical data to S1 Table. (DOCX)

S7 Table Numerical data to S2 Fig. (DOCX)
