# Supplementary material for: Novel Phenanthrene-Degrading Bacteria Identified by DNA-Stable Isotope Probing
Source: PLoS One. 2015 Jun 22;10(6):e0130846. doi: 10.1371/journal.pone.0130846 (PMC4476716; doi:10.1371/journal.pone.0130846)
Supplement: S1 Fig — Phylogenetic tree for the taxonomic location of the bacteria corresponding 219- and 241-bp TRFs. The tree is based on 16S rDNA sequence and produced by MEGA 5.0. 2. (PDF) [file pone.0130846.s001.pdf]

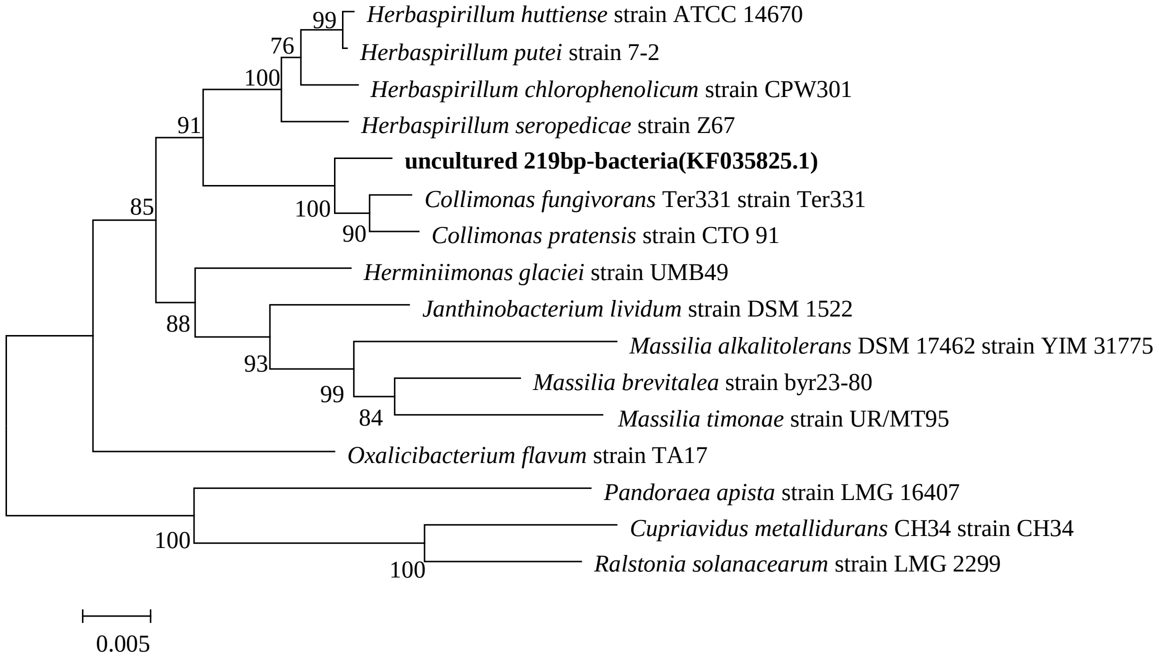

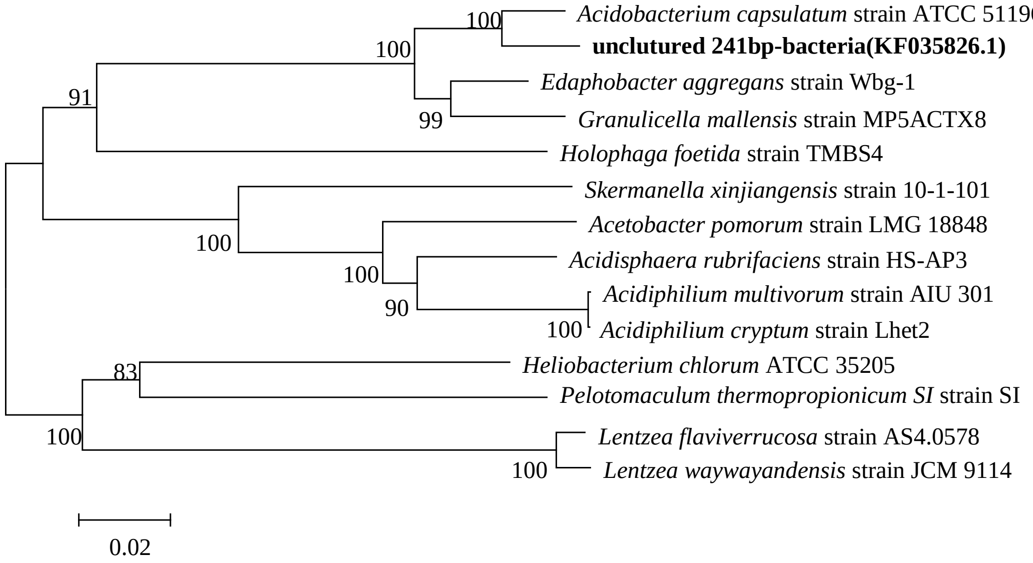

S1 Fig. Phylogenetic tree of the unclutured degraders.

Phylogenetic tree for the taxonomic location of the bacteria corresponding 219- and 241-bp TRFs. The tree is based on 16S rDNA sequence and produced by MEGA 5.0.
