# Supplementary material for: Novel Phenanthrene-Degrading Bacteria Identified by DNA-Stable Isotope Probing
Source: PLoS One. 2015 Jun 22;10(6):e0130846. doi: 10.1371/journal.pone.0130846 (PMC4476716; doi:10.1371/journal.pone.0130846)
Supplement: S2 Fig — The density distribution of total DNA in the micocosms amended with unlabled (12C) or labled (13C) PHE after centrifugation on days 3, 6 and 9. (PDF) [file pone.0130846.s002.pdf]

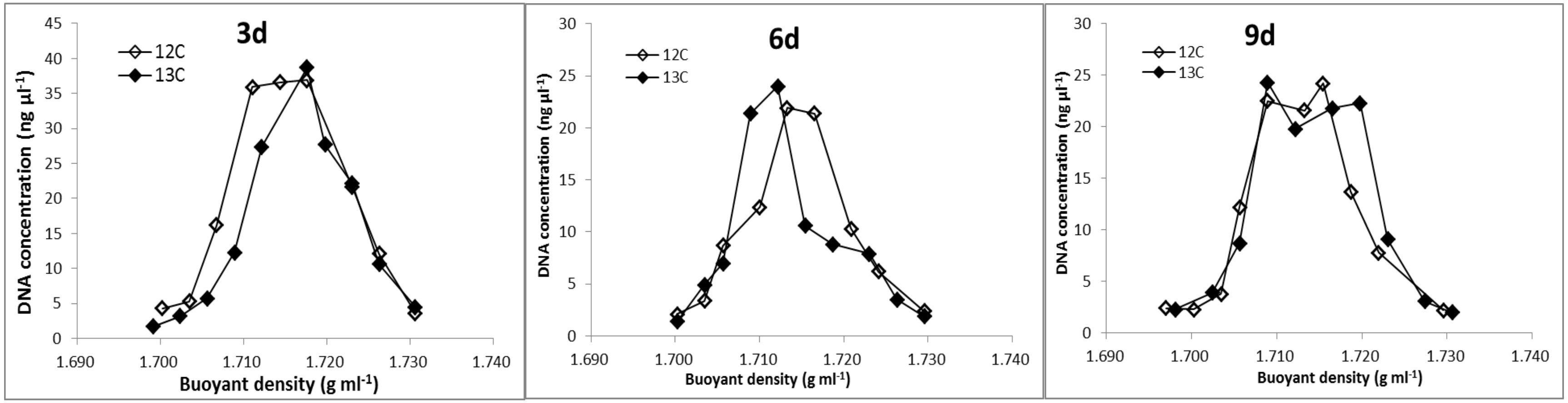

S2 Fig. The total DNA distribution in different buoyant desity.

The density distribution of total DNA in the micocosms amended with unlabled (^{12}C) or labled (^{13}C) PHE after centrifugation on days 3，6 and 9.
