# Supplementary material for: Novel Phenanthrene-Degrading Bacteria Identified by DNA-Stable Isotope Probing
Source: PLoS One. 2015 Jun 22;10(6):e0130846. doi: 10.1371/journal.pone.0130846 (PMC4476716; doi:10.1371/journal.pone.0130846)
Supplement: S1 Table — (DOCX) [file pone.0130846.s003.docx]

**S1 Table.** Phenanthrene degradation of isolates at 1 and 10 mg/kg in liquid media

| **Isolates** | **Phenanthrene degradation rate (%)** | |
| --- | --- | --- |
|  | 1 mg/kg | 10 mg/kg |
| Control (no isolate) | 8.5 ± 1.2 | 7.9 ± 0.9 |
| *Staphylococcus* sp. PHE-3 | 75.6 ± 8.2 | 38.2 ± 4.5 |
| *Pseudomonas* sp. PHE-1 | 98.5 ± 1.2 | 51.2 ± 3.7 |
| *Pseudomonas* sp. PHE-2 | 82.3 ± 9.0 | 27.4 ± 4.1 |
