# Supplementary material for: Novel Phenanthrene-Degrading Bacteria Identified by DNA-Stable Isotope Probing
Source: PLoS One. 2015 Jun 22;10(6):e0130846. doi: 10.1371/journal.pone.0130846 (PMC4476716; doi:10.1371/journal.pone.0130846)
Supplement: S2 Table — (DOCX) [file pone.0130846.s004.docx]

**S2 Table.** Primer pares used in the present study.

| Primer | Sequence (5’–3’) | References |
| --- | --- | --- |
| 27F | AGAGTTTGATCCTGGCTCAG | Luo et al., 2009 |
| 1492R | GGTTACCTTGTTACGACTT | Luo et al., 2009 |
| PAH-RHD_α_ GP F | CGGCGCCGACAAYTTYGTNGG | Cébron et al., 2008 |
| PAH-RHD_α_ GP R | GGGGAACACGGTGCCRTGDATRAA | Cébron et al., 2008 |
