# Supplementary material for: Novel Phenanthrene-Degrading Bacteria Identified by DNA-Stable Isotope Probing
Source: PLoS One. 2015 Jun 22;10(6):e0130846. doi: 10.1371/journal.pone.0130846 (PMC4476716; doi:10.1371/journal.pone.0130846)
Supplement: S3 Table — (DOCX) [file pone.0130846.s005.docx]

**S3 Table.** Numerical data to Fig 1 for T-RF 241bp.

| **T-RF 241bp** | **3d** | | **6d** | | **9d** | |
| --- | --- | --- | --- | --- | --- | --- |
| **^12^C** | **BD value(g/ml)** | **relative abundance(%)** | **BD value(g/ml)** | **relative abundance(%)** | **BD value(g/ml)** | **relative abundance(%)** |
|  | 1.700224 | 2.6872 | 1.700224 | 2.0384 | 1.696966 | 2.6709 |
|  | 1.703482 | 2.8402 | 1.703482 | 2.7874 | 1.700224 | 3.2704 |
|  | 1.706740 | 6.0407 | 1.705654 | 3.9065 | 1.703482 | 4.2780 |
|  | 1.711084 | 2.2997 | 1.709998 | 4.8000 | 1.705654 | 3.4340 |
|  | 1.714342 | 2.0259 | 1.713256 | 5.3852 | 1.708912 | 2.5000 |
|  | 1.717600 | 1.3880 | 1.716514 | 3.1448 | 1.713256 | 2.8183 |
|  | 1.723030 | 1.8180 | 1.720858 | 1.1008 | 1.715428 | 3.6511 |
|  | 1.726288 | 1.5338 | 1.724116 | 1.0388 | 1.718686 | 3.0000 |
|  | 1.730632 | 1.4028 | 1.729546 | 0.6542 | 1.721944 | 2.0640 |
|  |  |  |  |  | 1.729000 | 1.3800 |
| **^13^C** | 1.699138 | 2.0588 | 1.700224 | 1.1072 | 1.698052 |  |
|  | 1.702396 | 3.3078 | 1.703482 | 1.2512 | 1.702396 | 2.1791 |
|  | 1.705654 | 3.0600 | 1.705654 | 2.0659 | 1.705654 | 4.1320 |
|  | 1.708912 | 2.1492 | 1.708912 | 2.0000 | 1.708912 | 4.6487 |
|  | 1.712170 | 9.2970 | 1.712170 | 2.1306 | 1.712170 | 1.7863 |
|  | 1.717600 | 6.0000 | 1.715428 | 0.7311 | 1.716514 | 2.1429 |
|  | 1.719772 | 4.8180 | 1.718686 | 1.7444 | 1.719772 | 1.8563 |
|  | 1.723030 | 3.2716 | 1.723030 | 2.0000 | 1.723030 | 1.4541 |
|  | 1.726288 | 2.5819 | 1.726288 | 3.0000 | 1.727374 | 0.8197 |
|  | 1.730632 | 2.5107 | 1.729546 | 4.0000 |  |  |
|  |  |  | 1.732000 | 3.2000 |  |  |
