# Supplementary material for: Novel Phenanthrene-Degrading Bacteria Identified by DNA-Stable Isotope Probing
Source: PLoS One. 2015 Jun 22;10(6):e0130846. doi: 10.1371/journal.pone.0130846 (PMC4476716; doi:10.1371/journal.pone.0130846)
Supplement: S4 Table — (DOCX) [file pone.0130846.s006.docx]

**S4 Table: Numerical data to Fig 1 for T-RF 219bp.**

| **T-RF 219bp** | **3d** | | **6d** | | **9d** | |
| --- | --- | --- | --- | --- | --- | --- |
|  | **BD value(g/ml)** | **relative abundance(%)** | **BD value(g/ml)** | **relative abundance(%)** | **BD value(g/ml)** | **relative abundance(%)** |
| **^12^C** | 1.700224 | 2.2373 | 1.700224 | 3.7554 | 1.696966 | 3.0740 |
|  | 1.703482 | 3.6804 | 1.703482 | 3.8658 | 1.700224 | 0.1073 |
|  | 1.706740 | 4.0000 | 1.705654 | 6.0000 | 1.703482 | 2.7172 |
|  | 1.711084 | 6.2000 | 1.709998 | 7.0000 | 1.705654 | 3.1806 |
|  | 1.714342 | 4.3029 | 1.713256 | 10.105 | 1.708912 | 3.0000 |
|  | 1.717600 | 3.4842 | 1.716514 | 3.0000 | 1.713256 | 1.1574 |
|  | 1.723030 | 2.6752 | 1.720858 | 0.2700 | 1.715428 | 0.3905 |
|  | 1.726288 | 2.1725 | 1.724116 | 0.9695 | 1.718686 | 0.9018 |
|  | 1.730632 | 1.0000 | 1.729546 | 1.0000 | 1.721944 | 0.4154 |
|  |  |  |  |  | 1.726000 | 0.5000 |
| **^13^C** | 1.699138 | 0.4308 | 1.700224 | 3.1526 | 1.698052 | 0.2850 |
|  | 1.702396 | 0.8237 | 1.703482 | 1.6537 | 1.702396 | 0.5078 |
|  | 1.705654 | 1.0138 | 1.705654 | 1.8316 | 1.705654 | 2.0000 |
|  | 1.708912 | 0.8561 | 1.708912 | 3.0000 | 1.708912 | 1.0000 |
|  | 1.712170 | 3.3798 | 1.712170 | 6.0000 | 1.712170 | 3.0000 |
|  | 1.717600 | 2.0902 | 1.715428 | 10.000 | 1.716514 | 11.563 |
|  | 1.719772 | 1.8598 | 1.718686 | 18.443 | 1.719772 | 6.2113 |
|  | 1.723030 | 1.0515 | 1.723030 | 9.1919 | 1.723030 | 1.9417 |
|  | 1.726288 | 0.9216 | 1.726288 | 4.6586 | 1.727374 | 0.2822 |
|  | 1.730632 | 0.4369 | 1.729546 | 1.9193 |  |  |
