# Supplementary material for: Novel Phenanthrene-Degrading Bacteria Identified by DNA-Stable Isotope Probing
Source: PLoS One. 2015 Jun 22;10(6):e0130846. doi: 10.1371/journal.pone.0130846 (PMC4476716; doi:10.1371/journal.pone.0130846)
Supplement: S5 Table — (DOCX) [file pone.0130846.s007.docx]

**S5 Table:** **Numerical data to Table 1.**

| **Time (days)** | **Range of % phenanthrene in:** | | |  |
| --- | --- | --- | --- | --- |
|  | **Sterile controls** | **^12^C** | **^13^C** |  |
| **3** | 85,87,90 | 66, 69, 72 | 64, 68, 75 |  |
| **6** | 83,84,87 | 40, 44, 45 | 42, 43, 48 |  |
| **9** | 86,88,89 | 30, 35, 36 | 26, 30, 35 |  |
