# Supplementary material for: Novel Phenanthrene-Degrading Bacteria Identified by DNA-Stable Isotope Probing
Source: PLoS One. 2015 Jun 22;10(6):e0130846. doi: 10.1371/journal.pone.0130846 (PMC4476716; doi:10.1371/journal.pone.0130846)
Supplement: S6 Table — (DOCX) [file pone.0130846.s008.docx]

**S6 Table: Numerical data to S1 Table.**

| **Isolates** | **Phenanthrene degradation rate (%)** | |
| --- | --- | --- |
|  | 1 mg/kg | 100 mg/kg |
| Control (no isolate) | 7.5, 8.3, 9.7 | 6.9, 8.0, 8.8 |
| *Staphylococcus* sp. PHE-3 | 67.4, 70.6, 88.8 | 34.9, 37, 42.7 |
| *Pseudomonas* sp. PHE-1 | 88.5, 96.5, 110.5 | 48.0, 51.6, 54.0 |
| *Pseudomonas* sp. PHE-2 | 70, 85.6, 91.3 | 23.3, 28.9, 30.0 |
