# Supplementary material for: Novel Phenanthrene-Degrading Bacteria Identified by DNA-Stable Isotope Probing
Source: PLoS One. 2015 Jun 22;10(6):e0130846. doi: 10.1371/journal.pone.0130846 (PMC4476716; doi:10.1371/journal.pone.0130846)
Supplement: S7 Table — (DOCX) [file pone.0130846.s009.docx]

**S7 Table: Numerical data to S2 Fig.**

|  | **3d** | | **6d** | | **9d** | |
| --- | --- | --- | --- | --- | --- | --- |
| **^12^C** | **BD value(g/ml)** | **DNA concentration (ng/μl)** | **BD value(g/ml)** | **DNA concentration (ng/μl)** | **BD value(g/ml)** | **DNA concentration (ng/μl)** |
|  | 1.700224 | 4.3 | 1.700224 | 2.1 | 1.696966 | 2.4 |
|  | 1.703482 | 5.3 | 1.703482 | 3.4 | 1.700224 | 2.3 |
|  | 1.706740 | 16.2 | 1.705654 | 8.7 | 1.703482 | 3.8 |
|  | 1.711084 | 35.9 | 1.709998 | 12.4 | 1.705654 | 12.2 |
|  | 1.714342 | 36.6 | 1.713256 | 21.9 | 1.708912 | 22.5 |
|  | 1.717600 | 36.9 | 1.716514 | 21.4 | 1.713256 | 21.6 |
|  | 1.723030 | 21.6 | 1.720858 | 10.3 | 1.715428 | 24.2 |
|  | 1.726288 | 12.1 | 1.724116 | 6.2 | 1.718686 | 13.7 |
|  | 1.730632 | 3.6 | 1.729546 | 2.4 | 1.721944 | 7.8 |
|  |  |  |  |  | 1.729000 | 2.2 |
| **^13^C** | 1.699138 | 1.7 | 1.700224 | 1.4 | 1.698052 | 2.3 |
|  | 1.702396 | 3.2 | 1.703482 | 4.9 | 1.702396 | 3.9 |
|  | 1.705654 | 5.7 | 1.705654 | 7 | 1.705654 | 8.7 |
|  | 1.708912 | 12.3 | 1.708912 | 21.4 | 1.708912 | 24.3 |
|  | 1.712170 | 27.4 | 1.712170 | 24.0 | 1.712170 | 19.8 |
|  | 1.717600 | 38.7 | 1.715428 | 10.6 | 1.716514 | 21.8 |
|  | 1.719772 | 27.7 | 1.718686 | 8.8 | 1.719772 | 22.3 |
|  | 1.723030 | 22.1 | 1.723030 | 7.9 | 1.723030 | 9.1 |
|  | 1.726288 | 10.6 | 1.726288 | 3.5 | 1.727374 | 3.1 |
|  | 1.730632 | 4.5 | 1.729546 | 1.9 | 1.730632 | 2.0 |
|  |  |  | 1.732000 | 1.4 |  |  |
